# Study on the changes in the microbiome before and after seed embryo after-ripening of *Fritillaria cirrhosa*

**DOI:** 10.3389/fpls.2025.1544052

**Published:** 2025-05-13

**Authors:** Wenshang Li, Can Zhao, Qian Tao, Weimin Zhang, Hai Wang, Guiqi Han, Zhuyun Yan

**Affiliations:** ^1^ State Key Laboratory of Southwestern Chinese Medicine Resources, Chengdu University of Traditional Chinese Medicine, Chengdu, China; ^2^ School of Pharmacy, Chengdu University of Traditional Chinese Medicine, Chengdu, China; ^3^ School of Medical Technology, Chengdu University of Traditional Chinese Medicine, Chengdu, China

**Keywords:** *Fritillaria cirrhosa*, seed embryo after-ripening, microbiome, seed germination, bacterial communities, fungal communities

## Abstract

**Introduction:**

Microorganisms play an important role in the embryonic development of plant seeds; however, there are no existing reports on the microbial communities associated with *Fritillaria cirrhosa* before and after embryo after-ripening.

**Methods:**

In this study, the microbial communities of *Fritillaria cirrhosa* seeds before and after after-ripening were analyzed using the Illumina MiSeq platform, targeting the V4–V5 region of the bacterial 16S rRNA gene and the ITS1 and ITS2 regions of fungal ribosomal RNA.

**Results:**

The results showed that bacterial communities were more susceptible to environmental stress and exhibited greater fluctuations compared to fungal communities, as reflected in higher diversity and significant changes in the relative abundance of dominant genera and species. After embryo after-ripening, the dominant fungal genera were *Botrytis* (SBAR, 29.35%), *Tetracladium* (SBAR, 15.86%), *Ilyonectria* (SBAR, 15.35%), and *Mrakia* (SBAR, 13.14%), while the dominant bacterial genera were *Pseudomonas* (SBAR, 26.69%) and *Stenotrophomonas* (SBAR, 16.30%).Prediction results suggested that the bacterial communities with sharply increased relative abundance after embryo after-ripening may interact with seeds through various pathways, including carbohydrate metabolism, absorption and utilization of nitrogen (N), sulfur (S), phosphorus (P), and iron (Fe), as well as secretion of antibiotics, vitamins, cytokinins, and amino acids. Functional validation revealed that most culturable fungi with sharply increased relative abundance had cellulase-degrading abilities, while most of the bacterial isolates were capable of absorbing and utilizing C, N, S, P, and Fe elements. Microbial co-occurrence network analysis indicated that the microbiome after embryo after-ripening formed an unstable, expansive, and rapidly changing network.

**Discussion:**

In summary, this study revealed the overall dynamics of the microbiome in *Fritillaria cirrhosa* seeds after embryo after-ripening and identified key microbial taxa exhibiting sharp shifts in relative abundance. This work provides a foundational understanding of the microbial succession associated with seed embryo after-ripening in *Fritillaria cirrhosa*, which may support seed after-ripening and germination, and enhance seed stress resistance.

## 1 Introduction

*Fritillaria cirrhosa* D. Don(Family, Liliaceae) is a perennial bulbous plant in the Liliaceae family and one of the source plants for the precious traditional Chinese medicine Fritillaria (Chuanbeimu). *F. cirrhosa* has various medicinal properties, including antitussive, expectorant, bronchodilatory, anti-inflammatory, antioxidant, and antitumor effects, and is commonly used to treat coughs in clinical practice. It holds a promising future in the pharmaceutical and healthcare markets (Dai et al., 2024). The annual demand for *F. cirrhosa* exceeds 2,000 tons in regions such as China, Korea, Japan, Canada, Australia, and Europe, with the price of raw plant materials reaching as high as 560 USD per kilogram (Cunningham et al., 2018).

*F. cirrhosa* grows in harsh environments and has a slow growth rate, taking 4–5 years to harvest bulbs. It is mainly distributed on the southern edge of the Qinghai-Tibet Plateau at altitudes above 3,000 meters (Ma, 2015; Chandora et al., 2023). Due to overharvesting and habitat destruction, wild resources of *F. cirrhosa* have significantly decreased, and it has become an endangered species (Xiong et al., 2020; Rohit et al., 2021). However, the majority of commercial medicinal materials still come from wild sources (Guo and Sun, 2015; Shi et al., 2023). At present, *F. cirrhosa* has started to be cultivated artificially, but large-scale artificial cultivation depends critically on seed propagation technology and bulb cultivation (Liu et al., 2013). However, when the seeds of *F. cirrhosa* mature and fall to the ground, the embryos are underdeveloped, exhibiting both morphological and physiological dormancy characteristics (Hu, 2008; Yv et al., 2008). The seeds collected show inconsistent maturity, and the duration of the embryo after-ripening process varies, which greatly limits the reproduction and cultivation of the seeds (Li and Han, 2021). Currently, people complete the after-ripening process of *F. cirrhosa* seeds through low-temperature soil or sand storage treatments, which somewhat improve the seed germination rate, but these methods are time-consuming, difficult to manage, and prone to soil-borne diseases (Xiong et al., 2020; Zhou et al., 2021).

The plant microbiome plays a crucial role in seed germination, growth, health, stress protection, and plant chemistry (Mendes and Raaijmakers, 2015). The seed microbiome is the origin of the plant microbiome, influencing seed germination and plant phenotype (Nelson, 2017), and plays a role in promoting seed health, seedling establishment, and overall plant vitality (Singh P et al., 2023). For example, culturable endophytes in rice seeds promote seed germination (Liu et al., 2024), and orchids acquire fungal carbon for symbiotic germination (Zhao et al., 2024). Different plant species have associated microbial communities during germination, and the interactions between plants and microorganisms are generally positive during germination (Eldridge et al., 2021). From 26 different plant species, endophytic fungi isolated from leaves and stem tissues significantly improved maize seed germination rates. Utilizing endophytes for seed priming has broad applications in seed technology and agricultural production (El-Nagar et al., 2024). However, little is known about the role of seed microbiomes during plant embryo after-ripening.

Existing research indicates that the interaction between *F. cirrhosa* and microorganisms influences the phenotypic plasticity of the plant (Wang et al., 2024). However, the succession of seed microbiomes before and after embryo after-ripening in *F. cirrhosa* seeds is not well understood, which limits the development of beneficial microorganisms for promoting seed germination and seedling establishment. Therefore, this study focuses on the structural characteristics of fungal and bacterial communities in the seed microbiome during embryo after-ripening. It aims to observe and describe the species composition and ecological functions of dominant microbial communities, predict and explore the role of bacterial communities with significant increases in relative abundance during embryo after-ripening, and investigate the correlation between co-occurrence network properties and community characteristics before and after embryo after-ripening. This research will provide an initial understanding of the complex ecological interactions and processes between the microbiome and seeds before and after embryo after-ripening.

## 2 Materials and methods

### 2.1 Sample collection and preparation

NiOn September 30, 2022, a batch of *F. cirrhosa* fruit with dark yellow or yellow-brown, unopened fruit skins was selected from the wild environment in Daofu County, Ganzi Prefecture, Sichuan Province (101°27′18.95″E, 30°30′30.83″N, ASL: 3379 m) as the source of seed samples. To avoid contamination from environmental microorganisms, surface sterilization of the collected fruit was performed according to the method by Yang et al (Yang et al., 2022), with some modifications. First, the fruit was rinsed at least three times with 50 mL of sterile distilled water. Next, the fruit was immersed in a 3% sodium hypochlorite solution for 2 minutes, followed by three washes with sterile distilled water. Then, the fruit was soaked in 75% ethanol for 25 seconds, followed by three washes with sterilized distilled water. To ensure the surface sterilization was complete, 150 µL of the last wash was spread on potato dextrose agar plates and incubated at 28°C for 72 hours. Finally, using sterile scalpels and tweezers, the seeds were carefully separated from individual fruits in a clean bench.

A portion of the seeds was placed in 10 mL sterile conical tubes and immediately frozen in liquid nitrogen, then stored in an ultra-low temperature freezer at -80°C for later use as the pre-after-ripening samples. Another portion of the seeds was wrapped in sterile gauze and buried in a sterile tissue culture bottle (300 mL) containing 150 g of washed and autoclaved river sand, with humidity controlled between 65% and 75%. Afterward, the seeds underwent the after-ripening treatment. First, they were placed in a constant temperature incubator at 15°C for 40 days to complete the morphological after-ripening process, with the seed development observed every 20 days. When the seed embryos were fully developed, they were transferred to a 4°C refrigerator for a 90-day physiological after-ripening process, to be used as post-after-ripening samples. To ensure seed germination potential, a random selection of samples before and after after-ripening was tested for seed vitality using the TTC (triphenyl tetrazolium chloride) method.

**Figure 1:**
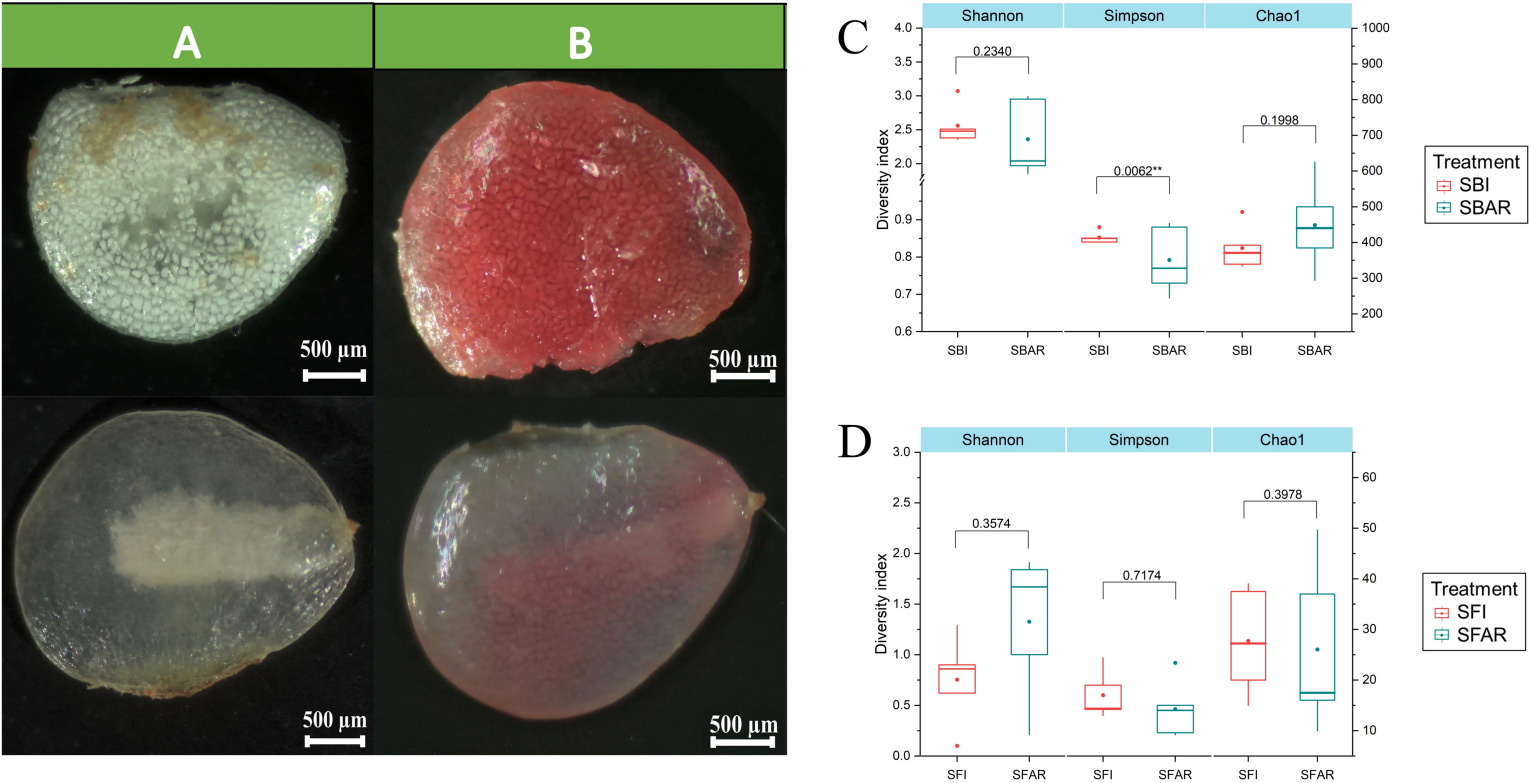
The morphology and vitality of seed embryos before and after after-ripening of *F. cirrhosa* seeds. **(A)** Seed morphology before and after embryo after-ripening. **(B)** Seed vitality staining before and after embryo after-ripening. Alpha diversity of bacterial **(C)** and fungal **(D)** communities before and after seed embryo after-ripening: before (SBI/SFI) and after (SBAR/SFAR). The left y-axis corresponds to the Shannon and Simpson indices, while the right y-axis corresponds to the Chao1 index. A two-sample t-test was used to compare alpha diversity differences before and after after-ripening. *P < 0.05; **P < 0.01. Treatments: SBI (SBI1–5), SBAR (SBAR1–5), SFI (SFI1–5), SFAR (SFAR1–5).

The experiment consisted of two treatments: after-ripening and non-after-ripening, with five repetitions for each treatment. Seed development was assessed by morphological observation (**Figure 1A**) and vitality analysis (**Figure 1B**). Seeds with well-developed embryos were selected as post-embryonic samples as well as samples without post-embryonic treatment as pre-embryonic samples for DNA extraction, respectively. The resulting DNA samples were divided into two portions for subsequent high-throughput fungal and bacterial testing. The DNA samples for fungal and bacterial community testing before and after after-ripening were labeled as Seed fungi not immature (SFI 1–5) and Seed fungus after ripening (SFAR 1–5) for fungi, and Seed bacterial immaturity (SBI 1–5) and Seeds bacterial after ripening (SBAR 1–5) for bacteria.

### 2.2 Genomic DNA extraction, PCR amplification, and sequencing

Seed samples from before and after embryo after-ripening were ground using autoclaved mortar and pestles. The microbial genomic DNA from each sample was extracted using the Omega Soil DNA Kit (Guangzhou Feiyang Bioengineering Co., Ltd.) according to the manufacturer’s instructions. DNA concentration and purity were assessed using a NanoDrop 2000 spectrophotometer, and the quality and integrity were further evaluated by 1% agarose gel electrophoresis. The DNA samples were stored at -20°C until further analysis.

For PCR amplification, two-step polymerase chain reaction (PCR) was used to generate the 16S rRNA and internal transcribed spacer (ITS) amplicons. The bacterial 16S rRNA gene V4-V5 region was amplified using the universal primers 515F and 806R (Caporaso et al., 2011), and the ITS region was amplified using the ITS1F and ITS2R primers (Toju et al., 2012). The PCR amplification used the TransGen AP221-02: TransStart Fastpfu DNA Polymerase in a 50 µL reaction system. Each sample was amplified in triplicate in 50 µL reaction tubes, containing 5 µL of template DNA (40–60 ng), 10 µL of 5× TransEco FastPfu Buffer, 5 µL of 2.5 mM dNTPs, 2 µL of Forward Primer (10 μM), 2 µL of Reverse Primer (10 μM), and ddH2O to make up a final volume of 50 µL. The PCR program consisted of an initial denaturation at 98°C for 3 minutes, followed by 32 cycles of denaturation at 98°C for 10 seconds, annealing at 78°C for 10 seconds, annealing at 55°C for 30 seconds, and extension at 72°C for 60 seconds. For ITS PCR amplification, the same procedure was followed, but without the PNA annealing step.

PCR products were purified by 2% agarose gel electrophoresis and the TruSeq^®^ DNA PCR-Free Sample Preparation Kit (Illumina, San Diego, CA, USA) was used to construct the DNA library. The DNA library was quantified using the QuantiFluor™-ST blue fluorescence quantification system (Promega), and the qualified DNA library was sent to Shanghai Meiji Biomedicine Technology Co., Ltd. for high-throughput sequencing using the Illumina Miseq platform (USA).

### 2.3 Sequence data processing and bioinformatics analysis

Raw sequencing reads were quality controlled and filtered using the FASTP software (v0.20.0) (Chen et al., 2018). The paired-end sequencing data were merged based on the overlap using the FLASH software (v1.2.7) (Magoč and Salzberg, 2011). Chimeric sequences were removed and optimized sequences were obtained using the QIIME software (v1.9.1). The sequences were aligned with the Unite and Silva databases using the Blast Zone tool in TBtools software (v1.12) to generate abundance tables for fungi and bacteria based on the highest matching scores (Chen et al., 2020).

To assess diversity, the data was rarefied to the minimum sequence number of samples using the vegan package in R software. The resulting abundance data was used to calculate the Chao1, ACE, Coverage, Shannon, and Simpson indices. Principal coordinates analysis (PCoA) based on Bray-Curtis distance and permutation multivariate analysis of variance (PERMANOVA) using the vegan package were applied to cluster the fungal and bacterial community structures before and after after-ripening. Statistical differences in microbial diversity were determined using two-sample t-tests in SPSS23.0 software (v23.0), with statistical significance set at p < 0.05.

The co-occurrence networks of fungal and bacterial communities before and after embryo after-ripening were constructed using Spearman correlation analysis (|r| > 0.8, p < 0.05). The correlation coefficients (r-values) and p-values were calculated using the psych package in R. Non-significant correlations (p > 0.05) were removed, and relationships with correlation coefficients greater than 0.8 were retained. The network nodes and edges were visualized using the Gephi software (v0.10.1) after constructing the network files using the igraph package. The PICRUSt2 software was used to predict the functional potential of bacterial communities with a significant increase in relative abundance, based on the 16S rRNA sequencing data and available microbial genome information.

### 1.4 Isolation, purification, and functional verification of the microbiome after seed embryo maturation

Seeds from the post-embryo maturation samples were placed into a sterile mortar, with a small amount of sterile water and sterile glass beads for grinding. After standing for 2 minutes, the supernatant was collected and spread onto NB and PDA agar plates. The control group consisted of the final wash liquid, and the plates were incubated at 37°C and 28°C in a constant temperature incubator. The plate streaking method was used to observe the colony morphology of the isolated strains. Single colonies with different morphologies were selected and streaked onto fresh agar plates to ensure the isolation of pure strains. The purified strains were then transferred to test tubes and incubated until growth was visible, after which they were stored at 4°C in a medical refrigerator. The fungal mycelium of the pure strains and bacterial colonies in the logarithmic phase were subjected to genomic DNA extraction and PCR amplification, and the samples were sent to Shanghai Meiji Bio-Medical Technology Co., Ltd. for sequencing. Fungal PCR amplification primers: ITS1 (5’-TCCGTAGGTGAACCTGCGG-3’) and ITS4 (5’-TCCTCCGCTTATTGATATGC-3’). Bacterial PCR amplification primers: 27F (5’-AGAGTTTGATCCTGGCTCAG-3’) and 1492R (5’-TACCTTGTTACGACTT-3’). The base sequences obtained from sequencing were matched with the base sequences of the bacterial communities with sharply increased relative abundance after embryo maturation, in order to identify culturable target strains for functional verification. Each strain in each functional verification experiment was tested in triplicate.

The cellulase degradation function of the fungal strains was verified using the Congo red staining method on CMC-Na (Carboxymethyl cellulose sodium) agar (Siqi et al., 2020). The purified target strains were inoculated in a triangular pattern on CMC-Na agar plates. Each strain was tested in triplicate and incubated at 28°C in a constant temperature incubator. After visible colonies formed, 2 mL of 0.2% Congo red reagent was added to the plates for staining. The plates were gently shaken to ensure the reagent covered the entire surface, and left for 15 minutes. Afterward, the Congo red reagent was discarded, and 2 mL of 0.5% NaCl solution was added to decolorize the plates. The plates were gently shaken again and left for 15 minutes before the NaCl solution was discarded. If a transparent hydrolysis zone appeared around the colony, the strain was capable of producing cellulase. The larger the hydrolysis zone, the stronger the cellulase activity.

The bacteria primarily verified the phosphorus solubilization (Chen et al., 2021) and iron-chelating functions (Grobelak and Hiller, 2017). The verification of other ecological functions of culturable bacteria will not be elaborated here, as it is detailed in the results section. Three sterile circular filter papers were placed in a triangular pattern on organic phosphate agar and CAS detection medium. The bacterial suspension was applied to the filter papers until fully wetted. Each strain was tested in triplicate and incubated in an inverted position at 30°C in a constant temperature incubator for 3 to 7 days. For phosphorus-solubilizing bacteria, the formation of transparent halos around the colonies was observed, while for iron-chelating bacteria, the change in color of the surrounding medium from blue to orange was observed.

## 3 Results

### 3.1 Microbiome sequencing information and diversity analysis of seeds before and after embryo after-ripening

The ITS and 16S rRNA gene sequencing results showed that the sequencing coverage for all samples was greater than 99.99% (**Supplementary Table S1**), indicating that the sequencing information was sufficient to reveal the fungal and bacterial community composition in each sample. A total of 389,342 fungal and 338,163 bacterial sequencing reads were obtained from 20 DNA samples. These reads were clustered into 129 fungal operational taxonomic units (OTUs) and 1,504 bacterial OTUs at a 97% similarity threshold. From the 10 pre-after-ripening seed samples, 195,807 effective fungal sequences and 169,241 effective bacterial sequences were obtained, which were classified into 87 fungal and 987 bacterial OTUs. From the 10 post-after-ripening seed samples, 193,535 effective fungal sequences and 177,159 effective bacterial sequences were obtained, which were classified into 65 fungal and 873 bacterial OTUs. The Chao1 index for the seed microbiome indicated no significant difference in fungal and bacterial richness before and after embryo after-ripening (p > 0.05). The Simpson index, however, showed significant differences in bacterial diversity before and after after-ripening (p < 0.05), while fungal diversity remained unchanged (p > 0.05). These results suggest that bacterial diversity exhibited a more pronounced change compared to fungal diversity during the after-ripening process (**Figure 1C**).

### 3.2 Dominant species composition of microbiomes before and after seed embryo after-ripening

**Figure 2:**
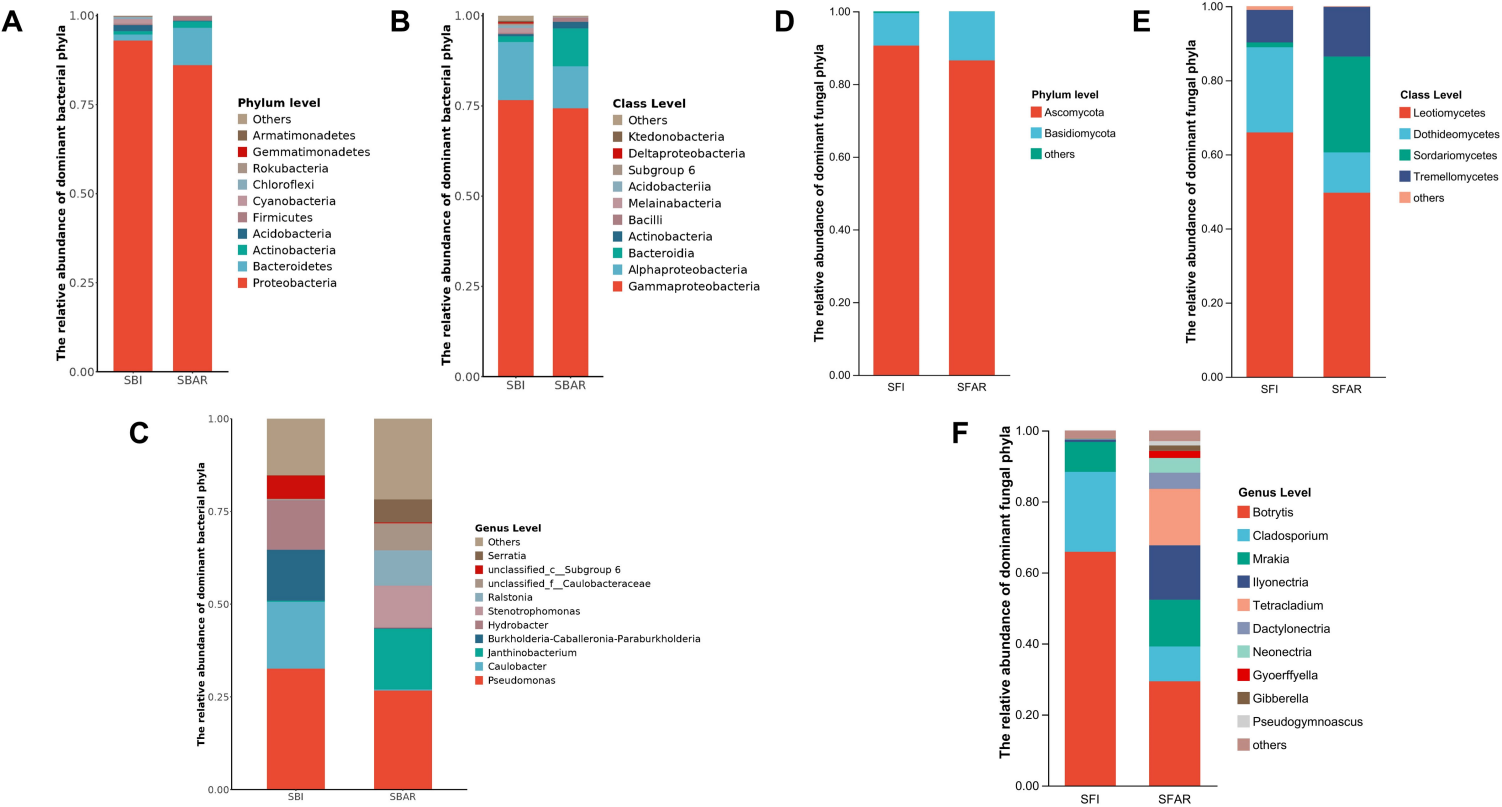
Relative abundance of dominant bacterial phyla **(A)**, classes **(B)**, and genera **(C)** before (SBI) and after (SBAR) seed embryo after-ripening, and dominant fungal phyla **(D)**, classes **(E)**, and genera **(F)** before (SFI) and after (SFAR) seed embryo after-ripening. Each bar represents the taxonomic composition of a sample either before or after after-ripening. Colors indicate different taxonomic categories (phyla, classes, and genera) within the bacterial and fungal communities. For both bacteria and fungi, five samples were collected before and after after-ripening, with sample IDs: SBI (SBI1–5), SBAR (SBAR1–5), SFI (SFI1–5), and SFAR (SFAR1–5).

The fungal community before after-ripening was classified into 4 phyla, 14 classes, 27 orders, 46 families, and 58 genera. Ascomycota and Basidiomycota were the dominant fungal phyla (**Figure 2B**). Leotiomycetes (SFI, 65.94%), Dothideomycetes (SFI, 22.99%), and Tremellomycetes (SFI, 8.78%) were the most abundant classes, with Botrytis (SFI, 65.83%), Cladosporium (SFI, 22.51%), and Mrakia (SFI, 8.45%) as the dominant genera. The fungal community after after-ripening was classified into 3 phyla, 11 classes, 19 orders, 37 families, and 49 genera. While the phyla remained the same, the class composition changed with Leotiomycetes (SFAR, 49.66%), Sordariomycetes (SFAR, 25.85%), and Tremellomycetes (SFAR, 13.42%) being dominant. Several genera with significant changes in relative abundance, such as Botrytis (SFAR, 29.35%), Tetracladium (SFAR, 15.86%), Ilyonectria (SFAR, 15.35%), Mrakia (SFAR, 13.14%), Cladosporium (SFAR, 9.83%), Dactylonectria (SFAR, 4.56%), and Neonectria (SFAR, 4.18%), Gyoerffyella(SFAR,1.98%), Gibberella(SFAR,1.54%), Pseudogymnoascus(SFAR,1.22%) were also observed (**Figure 2B**).

The bacterial community before after-ripening was classified into 22 phyla, 49 classes, 118 orders, 195 families, and 320 genera. Gammaproteobacteria (SBI, 76.61%) was the most abundant group, followed by Alphaproteobacteria (SBI, 16.11%). The dominant genera were Pseudomonas (SBI, 32.57%), Janthinobacterium (SBI, 18.10%), and Ralstoni (SBI, 13.79%) (**Figure 2A**). After after-ripening, the bacterial community was classified into 17 phyla, 47 classes, 114 orders, 181 families, and 313 genera. Gammaproteobacteria (SBAR, 74.38%) remained the most abundant group, but the Bacteroidetes class (SBAR, 10.56%) gradually became dominant. The bacterial community showed several genera with large changes in abundance proportions, Pseudomonas (SBAR,26.69%) was still the dominant genus, but the abundance of Janthinobacterium (SBAR,0.33%), and Ralstoni (SBAR,0.15%) was greatly reduced. Meanwhile Stenotrophomonas (SBI,0.25%; SBAR,16.30%), Luteibacter (SBI,0.09%; SBAR,9.51%), Tardiphaga (SBI,0.03%; SBAR,5.78%), Serratia (SBI,0.05%. SBAR, 11.14%), Chitinophaga (SBI, 0.03%; SBAR, 6.20%), and Massilia (SBI, 0.26%; SBAR, 7.35%) abundance increased significantly.

### 3.3 Community fluctuation of microbiomes before and after seed embryo after-ripening

**Figure 3:**
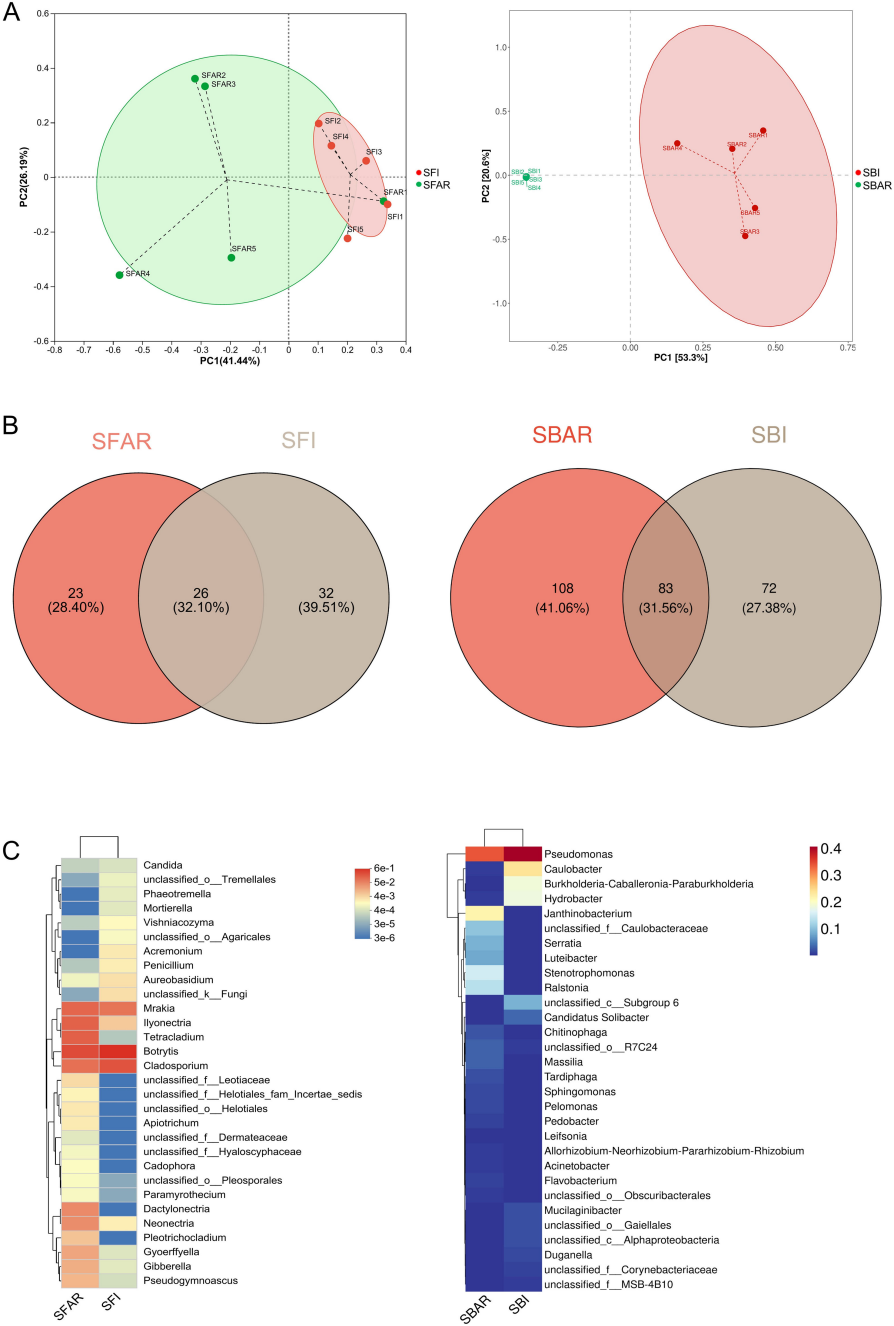
Principal component analysis (PCA) of bacterial samples before (SBI) and after (SBAR) seed embryo after-ripening and fungal samples before (SFI) and after (SFAR) **(A)**, OTU-based Venn diagrams **(B)**, and genus-level diversity heatmaps **(C)**, illustrating differences in species composition. Beta diversity analysis revealed that bacterial communities were more prone to fluctuations in response to environmental changes. Differences in the presence of shared and unique OTUs before and after seed embryo after-ripening were observed, which were reflected in both species composition and relative abundance. For both bacterial and fungal communities, five samples were collected before and after after-ripening. Sample IDs are as follows: SBI (SBI1–5), SBAR (SBAR1–5), SFI (SFI1–5), and SFAR (SFAR1–5).

PCoA (Principal Coordinates Analysis) results showed that, based on the after-ripening treatment, the bacterial community clustered more distinctly into two groups (“after-ripened treatment” and “non-after-ripened treatment”) compared to the fungal community (**Figure 3A**). PERMANOVA (permutation multivariate analysis of variance) results revealed that the bacterial community was more significantly affected by the after-ripening treatment (R² = 0.51464, P = 0.0090) than the fungal community (R² = 0.22715, P = 0.0051). The total number of OTUs at the genus level in the fungal community was 58 before after-ripening and 49 after after-ripening; in the bacterial community, the number of OTUs was 155 before after-ripening and 191 after after-ripening. After after-ripening, the fungal community had 23 new OTUs at the genus level, accounting for 28.40% of the total OTUs at the genus level, while the bacterial community had 108 new OTUs at the genus level, accounting for 41.06% of the total bacterial OTUs (**Figure 3B**).

There were significant increases in the relative abundance of OTUs shared between the pre- and post-after-ripening fungal and bacterial communities at the genus level (**Supplementary Tables S2**; **S3**). From these tables, OTUs that showed more than a tenfold increase in relative abundance before and after after-ripening were selected, as well as OTUs that newly appeared after after-ripening and had a relative abundance higher than the average of newly appearing species (328). The results indicated that in the post-after-ripening fungal community, 5 OTUs showed a more than tenfold increase in relative abundance, and 1 OTU newly appeared with a relative abundance higher than the threshold of 328; in the bacterial community, 17 OTUs showed a more than tenfold increase, and 9 OTUs newly appeared with a relative abundance higher than 328 (**Figure 3C**; **Supplementary Table S4**).

### 3.4 Functional prediction of bacterial communities with significant increases in relative abundance after seed embryo after-ripening

Functional prediction of bacterial communities with a significant increase in relative abundance after embryo after-ripening was performed using the PICRUSt2 tool, based on the 16S rRNA sequencing data and available microbial genome information. The results indicated that the functional roles of these bacterial communities primarily involved secondary metabolism across 11 categories (**Supplementary Figure 1**). The biosynthesis of secondary metabolites focused on compounds such as Prodigiosin biosynthesis, neomycin, kanamycin, carbapenems, streptomycin, tropane, piperidine and pyridine alkaloid biosynthesis, and isoquinoline alkaloid biosynthesis, as well as the degradation of various aromatic compounds. The microbial communities were also involved in the degradation of xenobiotics biodegradation and metabolism, such as cytochrome P450 and aromatic compound degradation pathways. Lipid metabolism was primarily involved in the biosynthesis of phospholipids for cellular membranes. Amino acid metabolism was associated with the biosynthesis and degradation of essential amino acids, including valine, leucine, isoleucine, aspartic acid, glutamic acid, and serine, with lesser contributions from tryptophan, histidine, arginine, and methionine metabolism. The metabolism of cofactors and vitamins involved the synthesis of vitamin H, vitamin B9, B1, B5, B2, retinol, and lipoic acid. Terpene and polyketide metabolism focused on the biosynthesis of insect hormones, zeatin, limonene, and pinene degradation, as well as the biosynthesis of antibiotics such as ansamycins and vancomycin. Carbohydrate metabolism was centered around starch and sucrose metabolism, the citric acid cycle (TCA cycle), acetyl acid and dicarboxylate metabolism, inositol phosphate metabolism, and pyruvate metabolism. Energy metabolism pathways included photosynthesis, oxidative phosphorylation, carbon fixation pathways in prokaryotes, carbon fixation in photosynthetic organisms, nitrogen metabolism, sulfur metabolism, and methane metabolism.

### 3.5 Functional verification of culturable microbial communities after seed embryo maturation

The culturable fungal community after embryo maturation consisted of 7 fungal operational taxonomic units (OTUs). The ratio of OTUs in the culturable fungal community to those in the non-culturable fungal community was 1:8. A total of 2 phyla, 3 classes, 4 orders, 4 families, and 5 genera were identified, with Ascomycota and Basidiomycota being the dominant phyla in the culturable fungal community (**Supplementary Table S5**). Most of the culturable fungi exhibited cellulase degradation functions (**Supplementary Figure 2A**), although the hydrolysis zone was not prominent.

The base sequences obtained from sequencing were matched with the base sequences of the bacterial communities with sharply increased relative abundance after embryo maturation, and a total of 7 genera and 26 strains of culturable bacteria were obtained (**Supplementary Table S6**). Of these, 23 strains exhibited phosphorus solubilization activity, accounting for 88.46% of the culturable bacteria (**Supplementary Figure 2C**). 21 strains exhibited iron-chelating function, accounting for 80.77% of the culturable bacteria (**Supplementary Figure 2B**). 24 strains were nitrogen-fixing bacteria, making up 92.31% of the culturable bacteria. 12 strains were nitrifying bacteria, accounting for 46.15%. 23 strains were sulfur bacteria, accounting for 88.46%. Verification of the ecological functions of culturable bacteria is shown in **Supplementary Table S6**.

### 3.6 Co-occurrence network features of the seed microbiome before and after embryo after-ripening

The co-occurrence networks of the fungal and bacterial communities before and after embryo after-ripening were constructed based on Spearman correlation analysis (|r| > 0.8, p < 0.05). These networks were used to represent the direct and indirect interactions between microbial taxa in the seed microbiome, and to examine the ecological relationships, structure, and functionality of these communities. The network properties were assessed using metrics such as the number of nodes, number of edges, and average degree to reflect the network complexity. Network cohesion was evaluated using graph density, clustering coefficient, and network diameter. Positively correlated lines represent potential reciprocal symbiotic relationships between species, while negatively correlated lines represent potential antagonistic competitive relationships between species.

**Figure 4:**
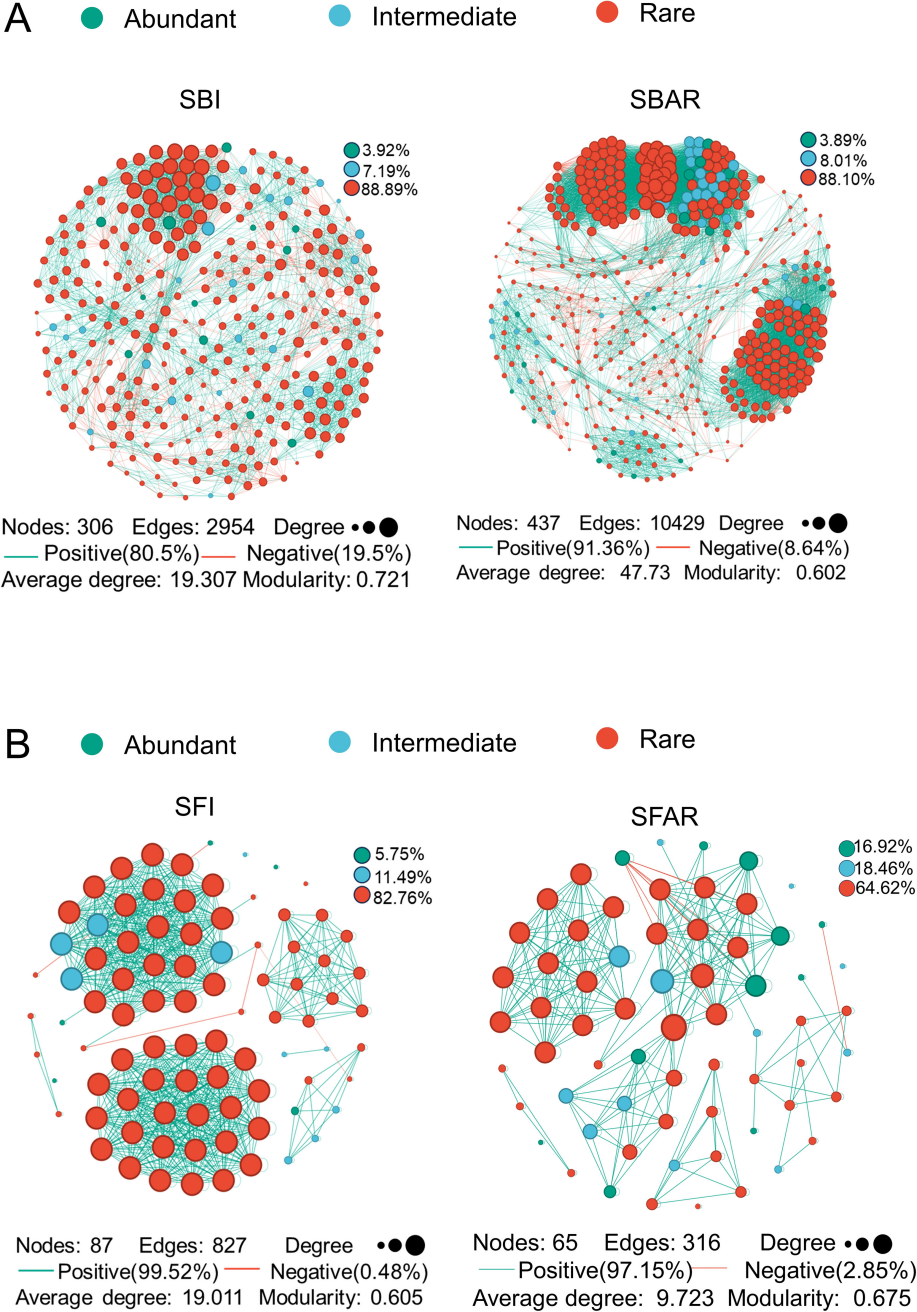
Bacterial network of pre-seed embryo maturation (SBI) and post-seed embryo maturation (SBAR) samples **(A)**, and fungal network of pre-seed embryo maturation (SFI) and post-seed embryo maturation (SFAR) samples **(B)**. All labels represent corresponding network properties, including the number of nodes, number of edges, relative abundance, proportion of positive correlations, proportion of negative correlations, average degree, and modularity coefficient. For both bacterial and fungal communities, five samples were collected before and after after-ripening. Sample IDs are as follows: SBI (SBI1–5), SBAR (SBAR1–5), SFI (SFI1–5), and SFAR (SFAR1–5).

The results showed that both the bacterial and fungal co-occurrence networks were composed primarily of low-abundance species (bacteria: SBI, 88.89%; SBAR, 88.10%; fungi: SFI, 82.76%; SFAR, 64.62%) (**Figures 4A, B**). In the bacterial network, the number of edges and network density increased by 353.05% after embryo after-ripening, while the modularity coefficient decreased by 16.50%, and the number of negative correlations decreased by 10.86%. In the fungal network, the number of edges and network density decreased by 61.79% after embryo after-ripening, while the modularity coefficient increased by 11.57%, and the number of negative correlations increased by 23.57%.

## 4 Discussion

The diversity and complexity of seed microbiomes are closely related to seed vitality and seed quality, and they influence various seed traits (Jarvis-Lowry et al., 2024; Santoyo et al., 2024). In this study, we described the diversity and complexity of the microbiome in *F. cirrhosa* seeds before and after embryo after-ripening, where changes in bacterial community diversity were more pronounced than in fungal communities. This finding indicates that the bacterial microbiome is more sensitive to the effects of after-ripening treatments (**Figures 1C**, **3C**). Previous studies have shown that bacteria in seeds generally have beneficial effects on seed development, while fungal communities in seeds may act as reservoirs for pathogens, potentially reducing seedling establishment without directly affecting seed vitality and germination during dormancy (Kabeere F and Hill, 1997).

The dominant fungal and bacterial communities before embryo after-ripening were primarily from the Ascomycota and Basidiomycota phyla for fungi, and the Gammaproteobacteria and Alphaproteobacteria classes for bacteria. These communities are also common in the rhizosphere microbiomes of wheat (Rossmann et al., 2020) and the seed microbiomes of Festuca sinensis in the Qinghai-Tibet Plateau (Gao et al., 2022). Before after-ripening, the dominant fungal genera were *Botrytis*, *Cladosporium*, and *Mrakia*. After after-ripening, additional genera such as *Tetracladium* and *Ilyonectria* appeared. The dominant bacterial genera before after-ripening were *Pseudomonas*, *Janthinobacterium*, and *Ralstoni*, while *Stenotrophomonas* and *Luteibacter* increased significantly after after-ripening. These genera, which have been observed in the seeds of monocots and dicots, may be considered core seed microbiome components.

*F. cirrhosa* seed germination is influenced by inhibitors and seed coat structures (Yv et al., 2008). After embryo after-ripening, the relative abundance of Botrytis and Cladosporium decreased. Botrytis is a facultative pathogen (Van Kan et al., 2014), while Cladosporium is a pathogenic genus that causes yellowing, necrotic lesions, and reduced root systems in plants (Salvatore et al., 2021). The dominant bacterial genus, Pseudomonas, showed little change in relative abundance, while Janthinobacterium decreased significantly, and Stenotrophomonas increased. Pseudomonas is a well-known plant growth-promoting bacterium (PGPB) that can directly or indirectly influence host plant growth, health, and development (Liu et al., 2012). Janthinobacterium, which can degrade organic and inorganic nitrogen sources to provide nitrogen for plants (Yang, 2019), was replaced by Stenotrophomonas, which contributes to the sulfur and nitrogen cycles (Kumar et al., 2023). Changes in the relative abundance of microbial taxa also reflect changes in microbial community structure and function. For example, the relative abundance of rice microbiome taxa significantly responded to signals such as the reduction of cadmium accumulation in contaminated soil when nitrate-carbon was added (Wu et al., 2023). Additionally, the stability of seed-associated microbial communities in response to invasion by phytopathogens varies, with some taxa being more stable under pathogen invasion, which is reflected in changes in relative abundance of related species (Rezki S et al., 2016). In our study, we selected OTUs from bacterial communities that exhibited significant increases in relative abundance before and after after-ripening (**Supplementary Table S4**) to explore the microbial metabolic functions involved in seed interactions. Functional predictions of bacterial communities indicated that these communities play a role in material cycling and energy flow during embryo after-ripening (**Supplementary Figure 1**). The conversion of substances during the after-ripening process is associated with changes in carbohydrate and protein content. Studies have shown that carbohydrate levels, peroxidase activity, and total soluble protein content reflect dormancy duration and the termination of specific proteins (Hobson and Davies, 1977; Kamenetsky et al., 2003; Koksal N and Eriş, 2011). In this study, predicted bacterial metabolic pathways were also associated with carbohydrate and protein metabolism, with energy flow processes involving photosynthesis and the cycling of carbon (C), nitrogen (N), and sulfur (S). Seeds also experience sulfur compound remobilization and redistribution, as well as nitrogen distribution and absorption during dormancy and early germination (Yasin and Bufler, 2007; Wei et al., 2021). These findings suggest a potential communication mechanism between bacteria and seeds, although further studies are needed to confirm these relationships (Mao et al., 2023).Therefore, we carried out the isolation, purification, and sequencing of the microbiome after embryo maturation, obtaining culturable target strains. Subsequently, we conducted ecological function verification of the culturable target strains after seed embryo maturation (**Supplementary Figure 2**). Most of the target strains possess one or more ecological functions, playing unique roles in plant growth and resistance to pathogens through the distribution and utilization of C, N, S, P, and Fe elements.

Microbial networks are an increasingly popular tool for studying microbial community structure because they integrate multiple types of information and can represent system-level behavior (Röttjers and Faust, 2018). In this study, bacterial co-occurrence networks were more tightly structured and complex after postembryonic ripening, but slightly less cohesive. The strength of reciprocal symbiotic interactions between bacterial community members was slightly enhanced (**Figure 4A**). The fungal network structure after embryo post-ripening was more sparse and cohesive with a slight increase in cohesion and a slight decrease in the strength of reciprocal symbiotic interactions among fungal community members (**Figure 4B**). These results suggest that both bacterial and fungal co-occurrence networks after seed embryo postripening are unstable, swollen and rapidly changing networks. It has been shown that the instability of co-occurrence networks is related to changes in environmental stress (De Vries et al., 2018). In this study, the instability, expansion and rapid change of bacterial and fungal co-occurrence networks may be related to the impending germination phenomenon of seeds (Magaña Ugarte et al., 2024), and the community structure and function will change at any time in response to environmental stress.

## 5 Conclusion

This study provides new perspectives and data on the succession of the microbial community before and after seed embryo maturation in *Fritillaria cirrhosa*. We conducted a systematic analysis of the bacterial and fungal communities before and after embryo maturation using amplicon-based community analysis methods. Through this approach, we found that the diversity and complexity of bacterial communities before and after seed embryo maturation are more susceptible to fluctuations due to environmental changes, as evidenced by significant shifts in the relative abundance of dominant bacterial genera and associated species. The dominant fungal genera in *Fritillaria cirrhosa* exhibit cellulase-degrading abilities and potential pathogenicity, which may disrupt seed coat structure during the post-maturation stage to facilitate seedling growth. Most of the dominant bacterial genera are plant-growth-promoting bacteria that can directly or indirectly influence the growth, health, and development of the host plant. The bacterial communities with significantly fluctuating relative abundances may participate in the seed embryo maturation process through various means, including the allocation and utilization of elements such as C, N, S, P, Fe, and the secretion of metabolic products. Furthermore, the microbial network after embryo maturation is unstable, expanding, and rapidly changing, allowing seeds to quickly adapt to external environments and germinate at the appropriate time. Further research is needed to understand how to utilize this seed microbial community in seed development and enhance seed stress resistance.

## Data Availability

The original contributions presented in the study are included in the article/**Supplementary Material**. Further inquiries can be directed to the corresponding author/s.
