# Supplementary material for: Study on the changes in the microbiome before and after seed embryo after-ripening of Fritillaria cirrhosa
Source: Front Plant Sci. 2025 May 13;16:1544052. doi: 10.3389/fpls.2025.1544052 (PMC12106415; doi:10.3389/fpls.2025.1544052)
Supplement: Supplementary file 1 [file Table1.docx]

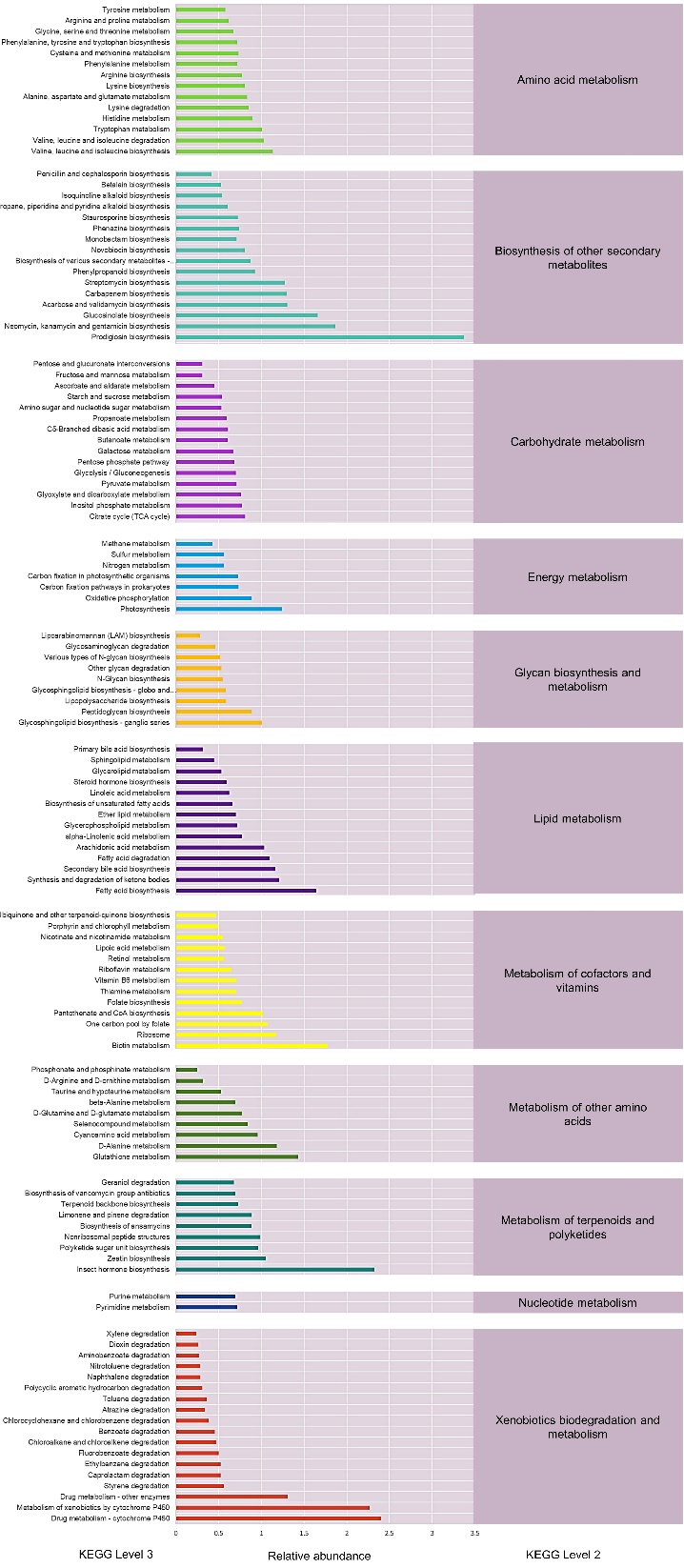


**Supplementary Figure 1.** Metabolic pathway analysis of bacterial communities with significantly altered relative abundance. The left side shows KEGG level 3 classifications, the center displays relative abundance, and the right side presents KEGG level 2 classifications.
