# Supplementary material for: Study on the changes in the microbiome before and after seed embryo after-ripening of Fritillaria cirrhosa
Source: Front Plant Sci. 2025 May 13;16:1544052. doi: 10.3389/fpls.2025.1544052 (PMC12106415; doi:10.3389/fpls.2025.1544052)
Supplement: Supplementary file 2 [file Table2.docx]

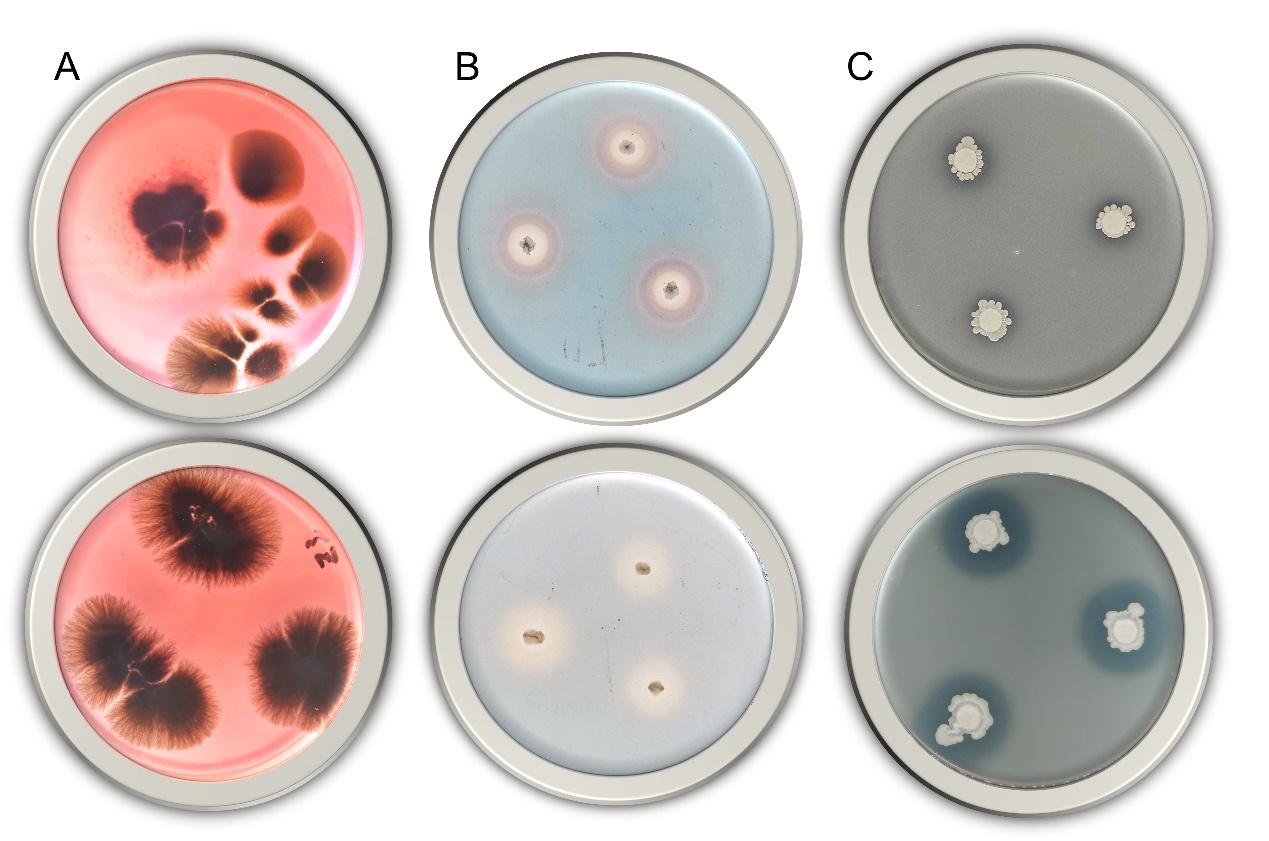


**Supplementary Figure 2.** Schematic diagram of functional verification of culturable microbial communities after seed embryo maturation. Fungal cellulase degradation function(A). Bacterial iron-chelating function(B). Bacterial phosphorus solubilization function(C). Each strain in each functional verification experiment was tested in triplicate.
