# Supplementary material for: Study on the changes in the microbiome before and after seed embryo after-ripening of Fritillaria cirrhosa
Source: Front Plant Sci. 2025 May 13;16:1544052. doi: 10.3389/fpls.2025.1544052 (PMC12106415; doi:10.3389/fpls.2025.1544052)
Supplement: Supplementary file 3 [file Table3.docx]

**Supplementary Table 1.** Diversity indices for each sample

|  | **Sample name** | **Sequence numbers** | **Coverage** | **Number of**  **OTUs** | **Alpha diversity** | | |
| --- | --- | --- | --- | --- | --- | --- | --- |
|  |  |  |  |  | **Shannon** | **Simpson** | **Chao1** |
| **Pre-after-ripening seed bacterial** | SBI1 | 34046 | 0.996138 | 303 | 2.380560 | 0.836962 | 392.2667 |
|  | SBI2 | 32823 | 0.996918 | 267 | 2.357850 | 0.839340 | 333.7255 |
|  | SBI3 | 35121 | 0.997400 | 302 | 2.509333 | 0.845949 | 339.1538 |
|  | SBI4 | 32074 | 0.996583 | 422 | 3.073788 | 0.880163 | 485.4242 |
|  | SBI5 | 35177 | 0.996658 | 304 | 2.476646 | 0.847939 | 370.7500 |
| **Post-after-ripening seed bacterial** | SBAR1 | 31802 | 0.994875 | 390 | 2.993373 | 0.881636 | 499.9186 |
|  | SBAR2 | 33225 | 0.993316 | 440 | 2.950196 | 0.885872 | 625.1724 |
|  | SBAR3 | 31618 | 0.997326 | 252 | 2.044935 | 0.686952 | 293.9016 |
|  | SBAR4 | 34150 | 0.995172 | 317 | 1.845037 | 0.726313 | 440.3088 |
|  | SBAR5 | 38127 | 0.995804 | 279 | 1.975631 | 0.772663 | 384.4667 |
| **Pre-after-ripening seed fungal** | SFI1 | 41435 | 0.995131 | 27 | 0.103567 | 0.973221 | 27.2000 |
|  | SFI2 | 43005 | 0.995914 | 14 | 0.896979 | 0.458912 | 20.0000 |
|  | SFI3 | 45348 | 0.996505 | 36 | 0.617933 | 0.700970 | 37.5000 |
|  | SFI4 | 33961 | 0.997589 | 39 | 1.291053 | 0.404271 | 39.0000 |
|  | SFI5 | 32058 | 0.995652 | 13 | 0.862341 | 0.472434 | 15.0000 |
| **Post-after-ripening seed fungall** | SFAR1 | 36115 | 0.994871 | 10 | 0.209202 | 0.921784 | 10.0000 |
|  | SFAR2 | 37790 | 0.994312 | 37 | 1.678111 | 0.238360 | 37.0000 |
|  | SFAR3 | 41533 | 0.996322 | 48 | 1.910242 | 0.206959 | 49.6666 |
|  | SFAR4 | 45935 | 0.997174 | 16 | 0.998959 | 0.498181 | 16.0000 |
|  | SFAR5 | 32162 | 0.992807 | 17 | 1.184466 | 0.448750 | 17.5000 |
