# Supplementary material for: Study on the changes in the microbiome before and after seed embryo after-ripening of Fritillaria cirrhosa
Source: Front Plant Sci. 2025 May 13;16:1544052. doi: 10.3389/fpls.2025.1544052 (PMC12106415; doi:10.3389/fpls.2025.1544052)
Supplement: Supplementary file 6 [file Table6.docx]

**Supplementary Table 4.** Composition of OTUs with a Dramatic Increase in Total RelativeAbundance Before and After Seed Embryo After-Ripening

| **Categorization** | **OTU** | **Phylum** | **Famil** | **Genus** | **GenuTotal relative abundance of OTUs before seed embryo after-ripening.** | **Total relative abundance of OTUs after seed embryo after-ripening.** |
| --- | --- | --- | --- | --- | --- | --- |
| **Fungal** | OTU87 | Ascomycota | Nectriaceae | Gibberella | 21 | 3123 |
|  | OTU121 | Ascomycota | Helotiaceae | Tetracladium | 22 | 31791 |
|  | OTU129 | Ascomycota | Nectriaceae | Ilyonectria | 1154 | 33750 |
|  | OTU115 | Ascomycota | Nectriaceae | Neonectria | 398 | 9423 |
|  | OTU126 | Ascomycota | Pseudeurotiaceae | Pseudogymnoascus | 58 | 2192 |
|  | OTU122 | Ascomycota | Melanommataceae | Pleotrichocladium | 0 | 1651 |
| **Bacterial** | OTU_19 | Proteobacteria | Xanthobacteraceae | Tardiphaga | 34 | 4723 |
|  | OTU_5 | Gammaproteobacteria | Xanthomonadaceae | Stenotrophomonas | 290 | 21832 |
|  | OTU_21 | Proteobacteria | Sphingomonadaceae | Pseudomonas | 17 | 2810 |
|  | OTU_10 | Proteobacteria | Enterobacteriaceae | Serratia | 64 | 14760 |
|  | OTU_2117 | Proteobacteria | Pseudomonadaceae | Pseudomonas | 48 | 2585 |
|  | OTU_6028 | Proteobacteria | Pseudomonadaceae | Pseudomonas | 9 | 894 |
|  | OTU_8 | Proteobacteria | Pseudomonadaceae | Pseudomonas | 459 | 8864 |
|  | OTU_17 | Proteobacteria | Burkholderiaceae | Massilia | 136 | 8725 |
|  | OTU_40 | Proteobacteria | Burkholderiaceae | Massilia | 54 | 821 |
|  | OTU_9 | Proteobacteria | Rhodanobacteraceae | Luteibacter | 117 | 12662 |
|  | OTU_51 | Actinobacteria | Microbacteriaceae | Leifsonia | 14 | 1042 |
|  | OTU_27 | Proteobacteria | Caulobacteraceae | Caulobacter | 70 | 1230 |
|  | OTU_37 | Proteobacteria | Beijerinckiaceae | Bosea | 5 | 495 |
|  | OTU_47 | Proteobacteria | Burkholderiaceae | Burkholderia-Caballeronia-Paraburkholderia | 32 | 394 |
|  | OTU_5765 | Proteobacteria | Rhizobiaceae | Ensifer | 7 | 413 |
|  | OTU_58 | Bacteroidetes | Sphingobacteriaceae | Pedobacter | 2 | 1389 |
|  | OTU_36 | Bacteroidetes | Sphingobacteriaceae | Pedobacter | 3 | 1384 |
|  | OTU_5859 | Proteobacteria | Xanthobacteraceae | Tardiphaga | 0 | 631 |
|  | OTU_96 | Firmicutes | Paenibacillaceae | Paenibacillus | 0 | 542 |
|  | OTU_62 | Actinobacteria | Micrococcaceae | Paenarthrobacter | 0 | 513 |
|  | OTU_224 | Bacteroidetes | Flavobacteriaceae | Flavobacterium | 0 | 911 |
|  | OTU_2678 | Bacteroidetes | Flavobacteriaceae | Flavobacterium | 0 | 903 |
|  | OTU_2095 | Proteobacteria | Xanthobacteraceae | Tardiphaga | 0 | 2426 |
|  | OTU_52 | Proteobacteria | Rhizobiaceae | Allorhizobium-Neorhizobium-Pararhizobium-Rhizobium | 0 | 1034 |
|  | OTU_1320 | Bacteroidetes | Flavobacteriaceae | Flavobacterium | 0 | 438 |
|  | OTU_206 | Firmicutes | Paenibacillaceae | Paenibacillus | 0 | 373 |
