# Supplementary material for: Study on the changes in the microbiome before and after seed embryo after-ripening of Fritillaria cirrhosa
Source: Front Plant Sci. 2025 May 13;16:1544052. doi: 10.3389/fpls.2025.1544052 (PMC12106415; doi:10.3389/fpls.2025.1544052)
Supplement: Supplementary file 7 [file Table7.docx]

| **Strain number** | **Phylum** | **Class** | **Order** | **Family** | **Genus** | **Cellulose degradation function** |
| --- | --- | --- | --- | --- | --- | --- |
| Z1 | Ascomycetes | Eurotiomycetes | Eurotiales | Aspergillaceae | Penicillium | Yes |
| Z2 | Basidiomycota | Agaricomycetes | Cystobasidiales | Cystobasidiaceae | Cystobasidium | Yes |
| Z3 | Ascomycetes | Dothideomycetes | Cladosporiales | Cladosporiaceae | Cladosporium | Yes |
| Z4 | Ascomycetes | Eurotiomycetes | Eurotiales | Aspergillaceae | Aspergillus | Yes |
| Z5 | Ascomycetes | Eurotiomycetes | Eurotiales | Aspergillaceae | Aspergillus | NO |
| Z6 | Ascomycetes | Eurotiomycetes | Eurotiales | Aspergillaceae | Aspergillus | Yes |
| Z7 | Basidiomycota | Tremellomycetes | Trichosporonales | Trichosporonaceae | Cutaneotrichosporon | NO |

**Supplementary Table 5.** Classification and function of culturable fungal communities
