# Supplementary material for: Study on the changes in the microbiome before and after seed embryo after-ripening of Fritillaria cirrhosa
Source: Front Plant Sci. 2025 May 13;16:1544052. doi: 10.3389/fpls.2025.1544052 (PMC12106415; doi:10.3389/fpls.2025.1544052)
Supplement: Supplementary file 8 [file Table8.docx]

**Supplementary Table 6.** Validation of ecological functions of culturable bacteria

| **Strain number** | **Categorize** | | | | | **Ecological Functions** | | | | | | | | | |
| --- | --- | --- | --- | --- | --- | --- | --- | --- | --- | --- | --- | --- | --- | --- | --- |
|  | **Phylum** | **Class** | **Order** | **Family** | **Genus** | **Ammoniation** | **Nitrogen fixation** | **Phosphate solubilization** | **Nitrification** | **Denitrification** | **Nitrosation** | **Cellulose decomposition** | **Vulcanization** | **Cyanobacteria (blueNogreen algae)** | **Siderophores** |
| B1 | Proteobacteria | Gammaproteobacteria | Enterobacteriales | Enterobacteriaceae | Serratia | No | Yes | Yes | No | Yes | Yes | Yes | Yes | No | Yes |
| B2 | Proteobacteria | Gammaproteobacteria | Enterobacteriales | Enterobacteriaceae | Serratia | Yes | Yes | Yes | No | No | No | No | Yes | No | Yes |
| B3 | Proteobacteria | Alphaproteobacteria | Rhizobiales | Rhizobiaceae | Ensifer | No | Yes | Yes | No | No | Yes | Yes | Yes | Yes | No |
| B4 | Proteobacteria | Alphaproteobacteria | Rhizobiales | Rhizobiaceae | Ensifer | Yes | No | Yes | No | No | No | Yes | No | Yes | Yes |
| B5 | Proteobacteria | Alphaproteobacteria | Rhizobiales | Rhizobiaceae | Ensifer | Yes | Yes | Yes | No | No | Yes | Yes | Yes | No | No |
| B6 | Proteobacteria | Alphaproteobacteria | Rhizobiales | Rhizobiaceae | Ensifer | Yes | Yes | Yes | No | No | Yes | Yes | Yes | No | Yes |
| B7 | Proteobacteria | Gammaproteobacteria | Xanthomonadales | Xanthomonadaceae | Stenotrophomonas | Yes | Yes | Yes | No | Yes | No | Yes | Yes | Yes | Yes |
| B8 | Bacteroidetes | Bacteroidia | Flavobacteriales | Flavobacteriaceae | Flavobacterium | Yes | Yes | Yes | No | No | No | Yes | Yes | Yes | Yes |
| B9 | Proteobacteria | Gammaproteobacteria | Pseudomonadales | Pseudomonadaceae | Pseudomonas | Yes | Yes | Yes | Yes | Yes | Yes | Yes | Yes | Yes | Yes |
| B10 | Proteobacteria | Gammaproteobacteria | Pseudomonadales | Pseudomonadaceae | Pseudomonas | Yes | Yes | Yes | No | No | No | Yes | Yes | Yes | Yes |
| B11 | Proteobacteria | Gammaproteobacteria | Pseudomonadales | Pseudomonadaceae | Pseudomonas | Yes | Yes | Yes | Yes | Yes | No | No | Yes | Yes | Yes |
| B12 | Proteobacteria | Gammaproteobacteria | Pseudomonadales | Pseudomonadaceae | Pseudomonas | Yes | Yes | Yes | Yes | Yes | No | Yes | Yes | Yes | Yes |
| B13 | Proteobacteria | Gammaproteobacteria | Pseudomonadales | Pseudomonadaceae | Pseudomonas | Yes | Yes | Yes | No | No | No | No | Yes | Yes | Yes |
| B14 | Proteobacteria | Gammaproteobacteria | Pseudomonadales | Pseudomonadaceae | Pseudomonas | Yes | Yes | Yes | Yes | Yes | Yes | Yes | Yes | Yes | Yes |
| B15 | Proteobacteria | Gammaproteobacteria | Pseudomonadales | Pseudomonadaceae | Pseudomonas | Yes | Yes | Yes | Yes | Yes | Yes | Yes | Yes | Yes | Yes |
| B16 | Proteobacteria | Gammaproteobacteria | Pseudomonadales | Pseudomonadaceae | Pseudomonas | Yes | Yes | Yes | No | No | Yes | Yes | Yes | Yes | Yes |
| B17 | Proteobacteria | Gammaproteobacteria | Pseudomonadales | Pseudomonadaceae | Pseudomonas | No | Yes | Yes | Yes | Yes | Yes | Yes | Yes | Yes | No |
| B18 | Proteobacteria | Gammaproteobacteria | Pseudomonadales | Pseudomonadaceae | Pseudomonas | No | Yes | Yes | Yes | Yes | Yes | Yes | Yes | Yes | Yes |
| B19 | Proteobacteria | Gammaproteobacteria | Pseudomonadales | Pseudomonadaceae | Pseudomonas | No | Yes | Yes | Yes | Yes | Yes | Yes | Yes | Yes | Yes |
| B20 | Proteobacteria | Gammaproteobacteria | Pseudomonadales | Pseudomonadaceae | Pseudomonas | No | Yes | Yes | Yes | Yes | Yes | Yes | Yes | Yes | Yes |
| B21 | Proteobacteria | Gammaproteobacteria | Pseudomonadales | Pseudomonadaceae | Pseudomonas | Yes | Yes | Yes | Yes | Yes | Yes | Yes | Yes | No | Yes |
| B22 | Firmicutes | Bacilli | Bacillales | Paenibacillaceae | Paenibacillus | Yes | Yes | Yes | Yes | No | Yes | Yes | Yes | No | Yes |
| B23 | Proteobacteria | Gammaproteobacteria | Betaproteobacteriales | Burkholderiaceae | Variovorax | Yes | Yes | Yes | No | No | No | No | Yes | Yes | Yes |
| B24 | Bacteroidetes | Bacteroidia | Sphingobacteriales | Sphingobacteriaceae | Pedobacter | No | Yes | No | No | No | No | No | No | No | No |
| B25 | Proteobacteria | Alphaproteobacteria | Rhizobiales | Rhizobiaceae | Ensifer | Yes | Yes | No | Yes | No | No | Yes | Yes | Yes | Yes |
| B26 | Proteobacteria | Gammaproteobacteria | Pseudomonadales | Pseudomonadaceae | Pseudomonas | Yes | No | No | No | No | No | No | No | No | No |
